# The effects of contracting Covid-19 on cognitive failures at work: implications for task performance and turnover intentions

**DOI:** 10.1038/s41598-022-13051-1

**Published:** 2022-05-25

**Authors:** James W. Beck, Arden Flow

**Affiliations:** Department of Psychology, University of Waterloo, Waterloo, ON N2L 3G1 Canada

**Keywords:** Psychology, Human behaviour

## Abstract

Individuals who contract Covid-19 often experience problems with memory, attention, and concentration, even after recovering from the initial illness. In the current manuscript, we argue that these symptoms are likely to manifest as cognitive failures in the workplace. Downstream, cognitive failures were expected to be associated with decreased task performance and increased turnover intentions. We collected data from a sample of working adults who either had (*n* = 45) or had not (*n* = 49) contracted Covid-19 at least one month prior to the study. Both groups were matched on key demographic characteristics. As anticipated, individuals who had contracted Covid-19 reported significantly more cognitive failures at work, relative to individuals who did not. More so, having contracted Covid-19 had significant indirect effects on task performance and turnover intentions via cognitive failure. These results indicate that beyond physical harm, Covid-19 can also have a detrimental influence on an individual’s capacity to perform at work.

## Introduction

Since its emergence in December 2019, the novel coronavirus disease (Covid-19) has had profound effects on society, infecting global populations en masse and becoming a leading cause of death worldwide. Although death is arguably the most salient consequence of contracting Covid-19, there can also be serious implications for individuals who have ostensibly “recovered” from the disease. Specifically, it is estimated that approximately one-third of individuals who contract Covid-19 experience persistent problems with memory, concentration, and sustained attention^1–3^. These symptoms can persist for months following initial recovery, and can occur even among individuals who experienced relatively mild cases of Covid-19^4,5^. Importantly, these problems are likely to affect workplace behavior, as cognitive functioning is a primary determinant of job performance (e.g., Ref. [^6^]). Therefore, there is an urgent need to understand the effects that contracting Covid-19 can have on work outcomes.

In particular, it is clear that Covid-19 is going to be an ongoing part of life, at least for the foreseeable future. The Delta and Omicron variants have led to accelerated growth in infections, and the Omicron variant in particular appears to spread rapidly even among vaccinated and previously infected individuals^7^. Therefore, in the coming months and years it will be increasingly common for individuals to contract Covid-19, recover, and return to work. Given the neurological symptoms associated with Covid-19 that were noted above, we expect many of these individuals will struggle to readjust to the workplace. Specifically, we expect these neurological symptoms to manifest as *cognitive failures* at work.

Cognitive failures are defined as errors made in mental processes during the execution of work tasks that an individual can usually perform successfully^8^. There are three facets of cognitive failures: memory, attention, and action. Memory cognitive failures refer to instances in which an individual fails to recall relevant work-related information, attention cognitive failures refer to a failure to remain focused on task-relevant information, and action cognitive failures refer to a failure to enact the correct steps involved in completing a work task. These facets are highly correlated, such that the experience of one type of cognitive failure tends to be associated with the experience of others. To this end, in the current research we treat workplace cognitive failures as a unitary construct. In particular, given the well-documented physiological and cognitive symptoms associated with Covid-19, we predict that having contracted Covid-19 is associated with increased cognitive failures at work (H1).

Downstream, we expect cognitive failures to affect work outcomes. These predictions are drawn from the Job Demands-Resources (JD-R) model^9^. The central tenet of the JD-R model is that individuals use *resources* to offset the *demands* of their jobs. Job resources are defined as features that are conducive to helping the individual achieve work goals, whereas job demands are the physical, social, psychological, and organizational aspects of the job that require continued physical or psychological effort^10^. In particular, memory, attention, and the ability to execute complex actions in sequence are resources that workers use to manage job demands. To this end, we argue that cognitive failures represent a resource deficit. Importantly, previous research has shown that when job demands outpace resources individuals experience a range of negative outcomes^11^. In the current research we focus on two specific outcomes: task performance and turnover intentions.

First, we expect cognitive failures to be negatively related to task performance, which is defined as specific behaviors that contribute to an organization’s goals^12^. Importantly, previous research indicates that as job demands exceed job resources, task performance is negatively impacted^11^. In particular, previous research has demonstrated a negative relationship between cognitive failures and task performance^13^. Thus, we expect there to be a negative indirect effect of having contracted Covid-19 on task performance via cognitive failure (H2).

Second, the degree to which job demands outpace job resources is also associated with increased turnover intentions^14,15^. That is, the degree to which an individual believes that they do not possess the necessary resources to cope with their work demands is positively associated with the likelihood that the person will voluntarily leave their job. Therefore, we expect that experiencing high levels of cognitive failure at work will lead individuals to question the degree to which they are able to cope with the demands of their current job, and possibly consider alternatives. Formally, we predict there will be a positive indirect effect of having contracted Covid-19 on turnover intentions via cognitive failures (H3).

## Methods

### Participants and procedure

The current study was comprised of a screening survey and a multi-wave focal study. All procedures were carried out in accordance with relevant ethical guidelines and regulations. Informed consent was obtained from all study participants. This study was approved by the University of Waterloo’s Office of Research Ethics (#43391).

#### Screening survey

A screening survey was used to determine eligibility for the focal study. Participants were recruited from Amazon’s Mechanical Turk (MTurk) on July 21, 2021. To ensure high-quality responses, the survey was visible only to individuals who resided in the United States and had a 90% MTurk approval rating based on at least 500 HITs. We pre-screened individuals by asking them to report the number of hours they worked per week (not including MTurk), and to respond to two items designed to screen out non-human respondents (i.e., “bots”). Only individuals who worked full-time (> 30 h) and passed the bot-screening items were given access to the screening survey.

Next, participants responded to demographic questions about their age, gender, racial group, education, employment sector, job tenure, household income, work arrangement (e.g. working from home), and whether or not they had been diagnosed with Covid-19. Nine-hundred and ninety-four individuals completed this survey. These individuals were informed that depending on their responses, they may be invited back for a subsequent research study. Participants received $0.10 USD for completing the screening survey.

#### Focal study

Participants from the pre-screen survey comprised a pool of potential participants for the focal study. All participants who indicated having contracted Covid-19 at least one month prior to the survey (*n* = 98) were invited to participate in the focal study. Individuals who had contracted Covid-19 less than one month prior to the study (N = 25) were ineligible for participation because the focal measures required reflection on one’s work experience “over the last month.”

Because Covid-19 has not spread uniformly across demographic groups^16^, we matched participants who had contracted Covid-19 with participants who had *not* contracted Covid-19 on key demographic variables (age, gender, race, education, and income). We dichotomized each demographic variable to facilitate this process. Specifically, age was dichotomized as “under 40 vs. over 40,” gender was dichotomized as “male vs. female,” race was dichotomized as “white vs. non-white,” education was dichotomized as “no bachelor’s degree vs. bachelor’s degree,” and household income was dichotomized at approximately the U.S. median (“under $70,000–$79,999 vs. over $70,000–$79,999”). For example, for every participant who indicated having contracted Covid-19 who was under 40, female, non-white, with a bachelor’s degree and a household income above the U.S. median, we invited a participant who had *not* contracted Covid-19 with the *same* demographic profile. This process was repeated for each unique demographic profile. In particular, there were 29 unique combinations of demographic profiles among the 98 participants who had contracted Covid-19. Because there were more individuals who had *not* contracted Covid-19 than there were participants who had contracted Covid-19, we used a random number generator to select participants within demographic categories. Ultimately, this process identified 196 individuals (*n* = 98 Covid-19, *n* = 98 control) to be invited to participate in the focal study.

The focal study was split into three surveys to reduce common method variance^17^. The surveys were administered on Monday (July 26, 2021), Wednesday (July 28, 2021), and Friday (July 30, 2021) of the same workweek. However, the Wednesday survey contained measures of constructs that are not reported in this article; specifically, selection-optimization-compensation (SOC)^18^ and perceived organizational support (POS)^19^. These measures were collected to test additional hypotheses that ultimately were not supported and thus are not presented here for the sake of expedience. Nonetheless, we present these results in the supplemental online materials (SOM) S1. Additionally, job-related affective well-being^20^ was measured as part of the Monday survey, and organizational citizenship behaviors (OCB)^21^ were measured as part of the Friday survey. We also present results regarding these variables in the SOM. In short, including these exploratory variables in the model does not change the interpretations of the results presented in the current manuscript.

Participants completed the measure of cognitive failure on Monday, and on Friday participants reported self-rated task performance and turnover intentions. Participants were paid $0.50 for completing each survey, and a $1.00 bonus for completing both surveys. Each survey included attention check items embedded within the focal measures. Of the 196 individuals invited to participate, 108 provided complete data. Fourteen participants’ data were excluded from our analyses due to failed attention checks (*n* = 6) and duplicate responding (*n* = 8)^22^. Thus, the final sample consisted of 94 individuals (57.45% male) with a mean age of 38.69 (*SD* = 10.68). Most of the participants were White (*N* = 73). On average, participants had worked at their current job for 11.96 years (SD = 6.21).

Forty-five participants had been diagnosed with Covid-19, and the remaining 49 participants had not. Importantly, these groups were equivalent in terms of age (*t*(92) = 0.18, *SE* = 2.22, *p* = 0.861), gender (χ^2^(1) = 0.60, *p* = 0.440), race (χ^2^(6) = 9.86, *p* = 0.131), education (χ^2^(7) = 12.64, *p* = 0.081), and income (*t*(92) = -0.14, *SE* = 1.03, *p* = 0.893). These groups also reported equivalent tenure at their current job (t(89) = 1.53, SE = 1.30, p = 0.129) and were evenly distributed across employment sector (χ^2^(16) = 16.88, *p* = 0.393; see Table 1). Finally, these groups did not differ with regard to how Covid-19 affected their working arrangement (χ^2^(4) = 8.42, *p* = 0.077). In particular, participants were asked: “Did you switch to working from home because of Covid-19?” Response frequencies for this item are shown in Table 2.

**Table 1:** Number of participants per employment sector.

Label	Covid-19 = No		Covid-19 = Yes	
	Freq	Pct	Freq	Pct
Education and training	13	13.83	2	2.13
Business, management, and administration	6	6.38	6	6.38
Information technology	4	4.26	8	8.51
Health science	6	6.38	5	5.32
Finance	3	3.19	6	6.38
Transportation, distribution, and logistics	3	3.19	3	3.19
Marketing, sales, and service	2	2.13	3	3.19
Agriculture, food, and natural resources	3	3.19	1	1.06
Hospitality and Tourism	2	2.13	2	2.13
Manufacturing	2	2.13	2	2.13
Other	2	2.13	1	1.06
Law, public safety, corrections, and security	0	0.00	2	2.13
Real estate and land development	1	1.06	1	1.06
Science, technology, engineering, and mathematics	1	1.06	1	1.06
Arts, audio/video technology, and communication	1	1.06	0	0.00
Government and public administration	0	0.00	1	1.06
Human services	0	0.00	1	1.06

**Table 2:** Number of participants per work arrangement.

Response	Covid-19 = No		Covid-19 = Yes	
	Freq	Pct	Freq	Pct
Yes. I was not working from home before, but now I do.	20	21.28	16	17.02
No change. I was not working from home before, and I still do not work from home	12	12.77	19	20.21
No change. I have always worked from home	2	2.13	5	5.32
Opposite change. I was working from home before, but now I work at the office/job site	1	1.06	0	0.00
I did switch to working from home, but I have now returned to the office/job site	14	14.89	5	5.32

Participants responded to the item: “Did you switch to working from home because of Covid-19?”.

### Measures

#### Covid-19 diagnosis

We assessed whether participants had contracted Covid-19 by asking “Have you been diagnosed with Covid-19?” Participants either responded with “Yes, I have had Covid-19” or “No, I have not.” Individuals who responded “yes” were asked to select the approximate date they were diagnosed.

#### Cognitive failures

We assessed workplace cognitive failure with Wallace and Chen’s^8^ 15-item measure (α = 0.94). Participants responded on a scale of 1 (*never*) to 5 (*very often*), and were instructed to refer to the month leading up to study. Sample items included “I failed to recall work procedures,” “I was easily distracted by co-workers,” and “I accidentally dropped objects or things.”

#### Task performance

We collected self-ratings of task performance using Williams and Anderson’s^21^ seven-item measure (α = 0.84). Participants responded on a scale of 1 (*strongly disagree*) to 5 (*strongly agree*), and were instructed to refer to the month leading up to the study. A sample item included “I adequately completed assigned duties.”

#### Turnover intentions

We assessed turnover intentions with Kelloway et al.’s^23^ four-item measure (α = 0.95). Participants responded on a scale of 1 (*strongly disagree*) to 5 (*strongly agree*), and were instructed to refer to their current job. A sample item is “I am thinking about leaving this organization.”

### Analysis plan

All hypotheses were tested simultaneously in a single structural equation model. Variables were modeled as latent constructs, with the exception of the categorical Covid-19 variable, which was dummy coded (*no* = 0, *yes* = 1). We created item parcels to account for the relatively small sample size relative to the total number of items administered^24^. Specifically, three parcels were created as indicators of cognitive failure, with each parcel composed of the five items belonging to each sub-factor (memory, attention, action). For task performance, we computed an even-items parcel and an odd-items parcel. The same approach was used for turnover intentions. One of the turnover intention parcels had a small negative residual variance in the initial model, so this residual variance was restricted to zero. This change did not affect the interpretation of the results. Indirect effects were computed as the product of the relevant regression weights, and indirect effects were tested for significance via bias corrected bootstrapped confidence intervals based on 5000 samples.

## Results

**Figure 1:**
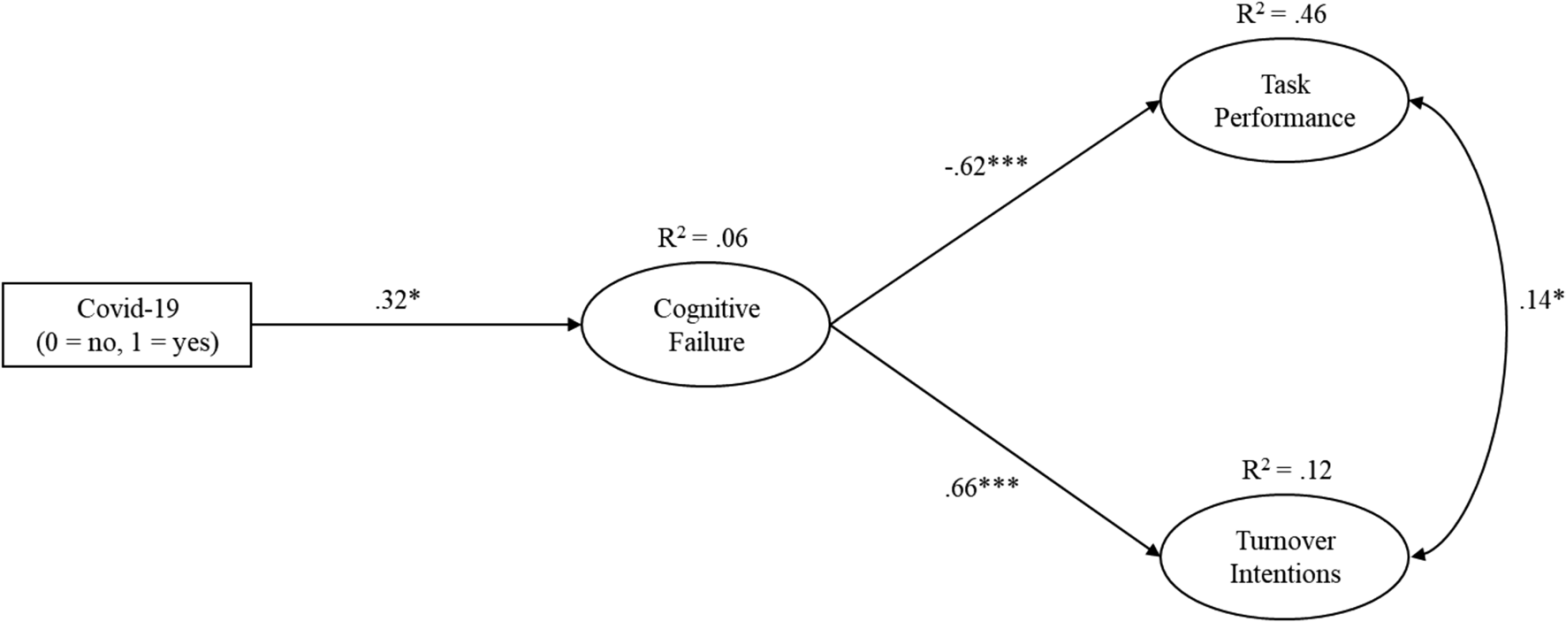
Structural model demonstrating the indirect effect of having contracted Covid-19 on task performance and turnover intentions via cognitive failure. *p < .05, ***p < .001.

Means, standard deviations, and intercorrelations are displayed in Table 3. The results from the test of the structural model are shown in Fig. 1. This model fit the data well (CFI = 0.993, RMSEA = 0.048, SRMR = 0.035). In support of H1, having contracted Covid-19 was positively related to cognitive failures (*b* = 0.32, *SE* = 0.15, *t* = 2.13, *p* = 0.034). Downstream, cognitive failures were negatively related to task performance (*b* = *− *0.62, *SE* = 0.11, *t* = − 5.54, *p* < 0.001) and positively related to turnover intentions (*b* = 0.66, *SE* = 0.17, *t* = 3.82, *p* < 0.001). Furthermore, there was a significant negative indirect effect of having contracted Covid-19 on self-rated job performance (effect = − 0.20, 95% CI [− 0.38, − 0.02]), providing supported for H2. Likewise, H3 was also supported, as there was a significant positive indirect effect of having contracted Covid-19 on turnover intentions (effect = 0.21, 95% CI [0.03, 0.45]).

**Table 3:** Means, standard deviations, and intercorrelations between latent variables.

	Mean	SD	1	2	3	4
1. Covid-19 (0 = no, 1 = yes)	0.48	0.50	1.00			
2. Cognitive Failure	1.80	0.69	0.23*	1.00		
3. Task Performance	4.52	0.60	− 0.16	− 0.68***	1.00	
4. Turnover Intentions	2.66	1.31	0.08	0.35***	− 0.06	1.00

N = 94. *p < 0.05, ***p < 0.001.

We also conducted two sets of auxiliary analyses. First, we assessed the degree to which participants’ work arrangement (see Table 2) affected the results of the hypothesis tests. To do so, we included the work arrangement item as a moderator of the relationships presented above. Importantly, this item was treated as a categorical variable in these analyses. Furthermore, the category “Opposite change. I was working from home before, but now I work at the office/job site” was dropped, as this category included only one participant. These results indicated that work arrangement had no influence on the results. Specifically, work arrangement did not moderate the relationship between Covid-19 and cognitive failure (*F*(3,85) = 1.94, *MSE* = 0.39, *p* = 0.129). Likewise, this variable also did not moderate the relationship between cognitive failure and task performance (*F*(3,84) = 1.10, *MSE* = 0.24, *p* = 0.354), nor the relationship between cognitive failure and turnover intentions (*F*(3,84) = 0.44, *MSE* = 1.53, *p* = 0.722).

Next, we restricted the sample to individuals who had contracted Covid-19 and computed the correlation between the number of days that had elapsed since the diagnosis and cognitive failure. This was done to assess the degree to which cognitive failures dissipate over time following recovery from Covid-19. Specifically, if cognitive failures were most pronounced immediately after contracting Covid-19 and then decreased over time, there would be a negative correlation between the amount of time that had elapses since contracting Covid-19 and cognitive failures. However, there was no significant correlation between time elapsed and cognitive failure (*r* = 0.16, *p* = 0.280), despite the fact that there was considerable variance in the amount of time that had elapses since contracting Covid-19 (*M* = 230.18, *SD* = 128.86, *Min* = 40, *Max* = 526). These results provide initial evidence that cognitive failures following recovery from Covid-19 are persistent, at least over the period of time included in the current study. Nonetheless, longitudinal data are required to provide a more complete account of cognitive failure trajectories over time.

## Discussion

Many individuals who contract Covid-19 end up returning to the workforce upon recovery. However, approximately one-third of the individuals who contract Covid-19 report experiencing persistent cognitive impairments^2^. To this end, the current research demonstrated that individuals who had contracted Covid-19 reported lower task performance and higher intentions to leave their jobs, relative to individuals who had not contracted Covid-19. These effects were mediated by cognitive failures at work.

We suspect that because death is a relatively rare outcome among Covid-19 patients, many individuals believe they are likely to be largely unaffected by Covid-19 if they are infected. However, our results indicate that contracting Covid-19 can have practical implications for individuals’ everyday lives; particularly, their ability to function effectively at work. As such, it is possible that beyond harming one’s physical health, Covid-19 also poses risks to financial well-being. Thus, the current research highlights the continued importance of protecting oneself from Covid-19. Unfortunately, the current data cannot speak to one of the most effective means of protecting oneself against Covid-19—vaccination. The majority (80%) of individuals in the current study reported contracting Covid-19 prior to March 2021, before vaccination became widely available in the United States. Thus, future research should assess whether fully vaccinated individuals who experience breakthrough Covid-19 cases experience the same degree of cognitive failure as unvaccinated individuals.

Another limitation of the current study is that we only measured whether or not individuals had contracted Covid-19. That is, we did not collect information from individuals who had contracted Covid-19 regarding the severity of their symptoms, whether they had experienced other symptoms of “long Covid” (e.g., fatigue), and the length of any absences from work that were experienced as a result of contracting Covid-19. We measured Covid-19 this way because the primary purpose of the study was to compare individuals who had contracted Covid-19 to individuals who had not. Nonetheless, it is possible that these factors may influence the degree to which an individual who contracted Covid-19 experienced cognitive failures at work. Therefore, future research should consider how the specific circumstances surrounding an individual’s experience with contracting Covid-19 influence cognitive failures and other work outcomes.

Future research should also assess the degree to which specific job characteristics influence the degree to which work outcomes are affected by having contracted Covid-19. For instance, it is well documented that the strength of the relationship between general mental ability (GMA) and job performance is moderated by job complexity, such that the positive relationship between GMA and job performance is stronger for highly complex jobs, compared to low-complexity jobs^25^. In a similar vein, we expect that the degree to which work outcomes (e.g., task performance, turnover intentions) are affected by cognitive failures is likely to depend on job complexity. Cognitive failures may be an *inconvenience* for someone performing a low-complexity job, yet this person may still be able to cope with the demands of the job. On the other hand, cognitive failures may make it more-or-less *impossible* to complete highly complex work. By definition, highly complex jobs require individuals to attend to and remember large amounts of information. An inability to do so is likely to be highly detrimental to performance, and may leave an individual looking for other sources of employment.

It is also important to note that we are unable to unequivocally demonstrate that having contracted Covid-19 *caused* increases in cognitive failures. For one, it is possible that this relationship could have been driven by third-variable causes. To this end, we addressed as many such causes as possible by matching participants on key demographic variables. Furthermore, as shown in the SOM, controlling for affective well-being did not have any substantive effect on the results. Second, it is possible that individuals who are prone to experience cognitive failures may be more likely to contract Covid-19. That is, causality may be reversed. Although possible, we see this as an unlikely explanation for our results. Nonetheless, it is important to acknowledge that random assignment cannot be achieved (individuals cannot be randomly assigned to contract Covid-19), meaning causal inferences will always be tempered to some degree.

Similarly, the use of a self-report measure of cognitive failures is another limitation of the current research. In particular, participants did not provide objective measures of cognitive functioning, such as tests of short-term memory and attention. Therefore, it is possible that having contracted Covid-19 led individuals to *believe* that they had experienced cognitive failures, even if there was no objective change in their memory, attention, or actions at work. For instance, individuals who consumed news coverage of “brain fog” attributable to Covid-19 may have been susceptible to experiencing similar symptoms, even in the absence of objective cognitive failures. Nonetheless, this limitation is reasonable for two reasons. First, there is meta-analytic evidence that contracting Covid-19 is associated with impaired memory and attention^2^. Second, workplace cognitive failures are most appropriately measured via self-reports, because individual workers have the most knowledge of their own day-to-day behaviors^26,27^.

To conclude, the current results may have important implications for managers and organizations more broadly. Individuals returning to work after contracting Covid-19 may experience difficulties returning to their pre-Covid-19 level of performance, and accommodations may be necessary. This may include reducing workloads, extending deadlines, or providing flexible work arrangements. By doing so managers may also be able to alleviate turnover intentions, as individuals will be less likely to feel their capacity to perform the job is outstripped by demands.

## Supplementary Information

Supplementary Information.

## Data Availability

The data presented in this article are available from the corresponding author upon request.
