# Supplementary material for: The effects of contracting Covid-19 on cognitive failures at work: implications for task performance and turnover intentions
Source: Sci Rep. 2022 May 25;12:8826. doi: 10.1038/s41598-022-13051-1 (PMC9130967; doi:10.1038/s41598-022-13051-1)
Supplement: Supplementary file 1 — Supplementary Information. [file 41598_2022_13051_MOESM1_ESM.docx]

**Supplemental Online Materials (SOM)**

We measured several variables that were not included in the submitted manuscript. First, we measured selection-optimization-compensation (SOC) and perceived organizational support (POS) as we believed these variables would moderate the indirect effect of Covid-19 on task performance and turnover intentions. Second, because all of the variables included in our model were self-reported, we measured affective well-being as a control variable. Finally, the task performance scale used in this research (Williams & Anderson, 1991) also contained items measuring organizational citizenship behaviors (OCB). In the sections below we present the results including these variables.

**Including SOC and POS as moderators**

We initially hypothesized that SOC would moderate the relationship between Covid-19 and cognitive failure. Specifically, we believed individuals who were highly adept and reshaping their tasks and compensating for their abilities (i.e., high SOC) would be better able to cope with the neurological effects of Covid-19, relative to individuals who exhibit less SOC. Likewise, we expected the detrimental effects of cognitive failure on task performance and turnover intentions to be attenuated by POS. For instance, individuals who perceived strong POS might received more accommodations, relative to individuals who perceived weaker POS.

We tested a structural model with SOC and POS as moderators. Because we were modeling interactions, we modeled manifest variables, rather than latent constructs (Maslowsky et al., 2015; Sardeshmukh & Vandenberg, 2017). All variables were transformed to z-scores in order to facilitate interpretations of the indirect effects. The did not fit the data well (CFI = .844, RMSEA = .140, SRMR = .065). Importantly, no interaction terms were significant. Furthermore, the main effects matched the results reported in the manuscript.


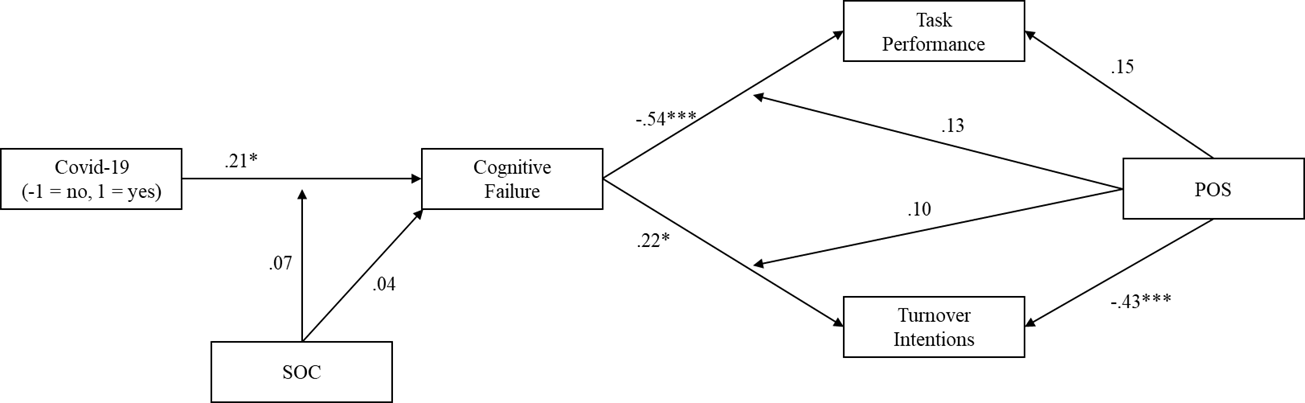


*Notes*: **p* < .05, ****p* < .001. SOC was allowed to covary with Covid-19, and both SOC and Covid-19 were allowed to covary with the interaction term. Cognitive failure and POS were allowed to covary, and both cognitive failure and POS were allowed to covary with the interaction term. Finally, task performance and turnover intentions were allowed to covary.

**Including affective well-being as a control variable**

Next, we included affective well-being as a covariate when testing the model presented in the manuscript. This model fit the data well (CFI = .960, RMSEA = .099, SRMR = .062). Importantly, none of the conclusions reported in the manuscript change when well-being is included. Thus, we report the more parsimonious model in the manuscript.


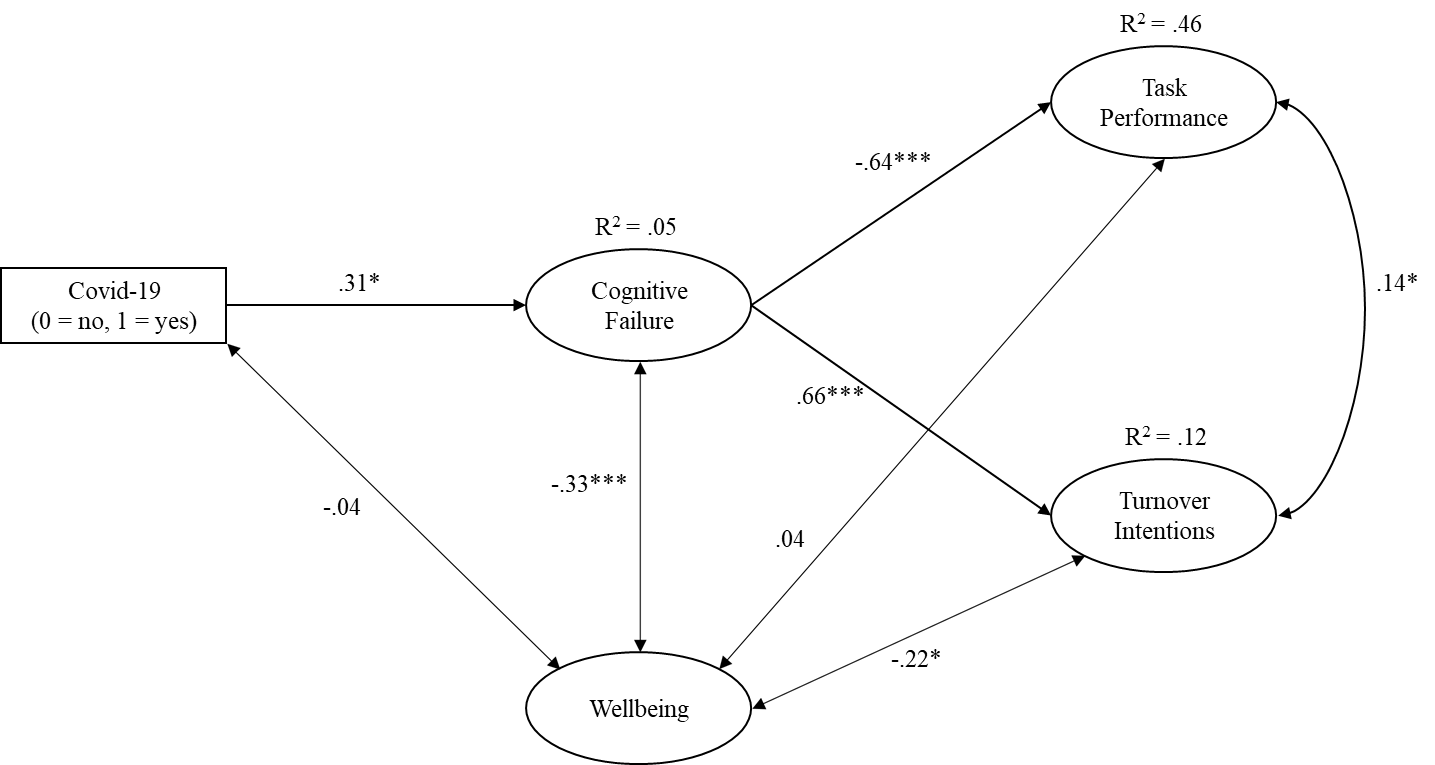


*Notes*: **p* < .05, ****p* < .001

**Including OCB as an additional dependent variable**

We did not have hypotheses regarding OCB, as we expected the effects of Covid-19 to be occur via cognitive mechanisms. Nonetheless, for the sake of completeness we also ran a model with OCB as an additional dependent variable. Williams and Anderson’s (1991) measure included items to measure OCBs directed toward individuals (OCB-I) and OCBs directed toward the organization (OCB-O). However, the three negatively worded OCB-O items included in this scale do not load with other OCB items, and instead are better characterized as counterproductive work behaviors (Henderson et al., 2020). Thus, we did not include these items in our analyses. Furthermore, given that OCB-I and OCB-O share large portions of variance, as well as common correlates (Podsakoff et al., 2009), and given the fact that we had no a priori hypotheses about OCB, we created a single OCB construct using the positively worded items. Although the model fit the data well (CFI = .977, RMSEA = .069, SRMR = .056), cognitive failures had no effect on OCBs. More importantly, the conclusions reported in the manuscript do not change.


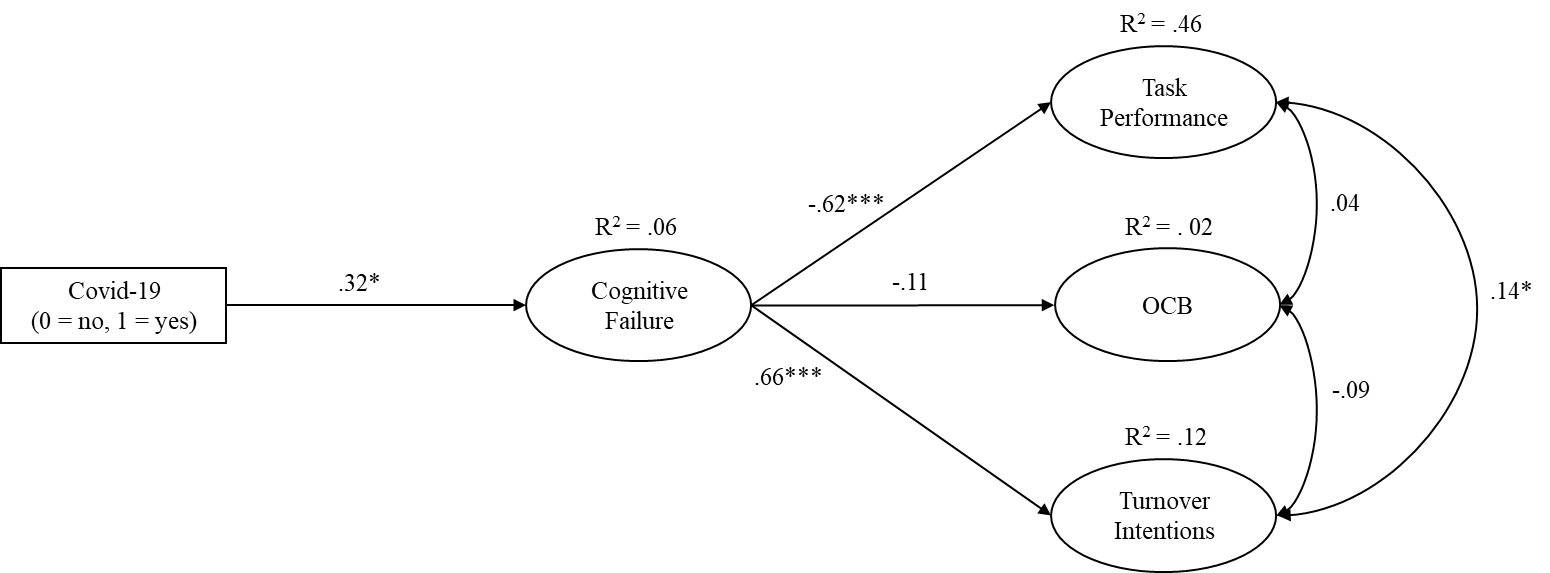


*Notes*: **p* < .05, ****p* < .001
